# Phenotypic and genetic changes in the life cycle of small colony variants of *Salmonellaenterica* serotype *Typhimurium* induced by streptomycin

**DOI:** 10.1186/s12941-016-0151-3

**Published:** 2016-05-31

**Authors:** Wanli Li, Yinghui Li, Yarong Wu, Yujun Cui, Yao Liu, Xiaolu Shi, Qian Zhang, Qiongcheng Chen, Qun Sun, Qinghua Hu

**Affiliations:** Key Laboratory of Bio-resources and Eco-environment of the Ministry of Education, College of Life Sciences, Sichuan University, 29# Wangjiang Rd., Chengdu, Sichuan 610064 P. R. China; Shenzhen Major Infectious Disease Control Key Laboratory, Shenzhen Centre for Disease Control and Prevention, Shenzhen, Guangdong 518055 P. R. China; State Key Laboratory of Pathogen and Biosecurity, Beijing Institute of Microbiology and Epidemiology, Beijing, 100071 P. R. China

**Keywords:** Small colony variants, *Salmonella*, Biofilm, *ubiE*, *prfB*

## Abstract

**Background:**

Small colony variants (SCVs), constituting a slow-growing subpopulation of bacteria that facilitates persistence in lethal environmental conditions, are able to revert to the phenotype of rapid growth for further proliferation and transmission. *Salmonella enterica* serotype *Typhimurium* is one of the most important foodborne pathogens. This study investigated the genetic mechanisms how SCVs induced by streptomycin reverted to the fast-growing phenotype and the phenotypic changes of SCVs among their complete life cycle in *S.Typhimurium*.

**Methods:**

*Salmonella Typhimurium* SCVs were obtained by streptomycin treatment and their revertants were collected in the absence of antibiotics. The fitness, antimicrobial susceptibility, biofilm formation, and the biofilm-related genes expression were analyzed in comparison to their wild type strain, and the whole genome sequencing was performed to identify the genetic changes in the life cycle of *S. Typhimurium* SCVs.

**Results:**

Small colony variants were characterized by an increased antimicrobial resistance to streptomycin (64-fold), imipenem (twofold), and gentamicin (fourfold). A significant increase in biofilm production with higher expression of *csgB* was observed in SCVs (*P* < 0.01). The genetic alterations of all SCVs occurred in *ubiE* gene (coenzyme Q_8_ and menaquinone synthesis) with frameshift mutations. However, all fast-growing revertants again lost the trait of increased biofilm production (*P* > 0.05), in which two modes of the genetic changes for reversing to the rapidly growing form were observed: four revertants harbored a secondary mutation in *ubiE*, which reinstated most of the amino acid sequence of the *ubiE*, and other four revertants harbored a mutation in *prfB*.

**Conclusions:**

*Salmonella Typhimurium* could switch to the phenotype of SCVs under the treatment of streptomycin by a mutation in *ubiE*, partially combined with increased production of biofilm, and these SCVs could escape from growth restriction by a compensatory mutation in *prfB* or a new mutation in *ubiE*. These findings may contribute to establishing phenotype-directed treatments against SCVs of *S.Typhimurium*.

**Electronic supplementary material:**

The online version of this article (doi:10.1186/s12941-016-0151-3) contains supplementary material, which is available to authorized users.

## Background

Small colony variants (SCVs), an aberrant form of bacterial phonotype, constitute a slow-growing subpopulation typically characterized with reduced metabolic activity and increased resistance to antibiotics [1, 2], thereby protecting bacterial cells against some non-optimal and lethal environmental conditions [3]. Incidences of SCVs have been reported in clinical specimens [4], livestock and even in foods [5].

Bacterial SCVs can be induced by cold stress [6], triclosan and living inside eukaryotic cells [1]. Additionally, exposure to antibiotics, like quinolones and aminoglycosides, has been shown to be able to induce bacterial SCVs [7, 8], resulting in an increased bacterial survival and ability to further proliferate. For example, streptomycin (STR), one of the aminoglycoside antibiotics, has been reported to induce SCVs of *S. aureus* [9]. STR, which was first used in plant agriculture in the 1950’s, remains to be an important antibiotic for bacterial disease control in both animals and plants, especially in developing countries [10, 11]. However, agricultural products, such as fruits, vegetables, livestock and poultry meats were found to be the most common sources of *Salmonella enterica* infections [12–14]. Therefore, *Salmonella* spp. SCVs may be induced by exposure to STR in agricultural products in these countries.

*Salmonella* spp. is a major causative agent of foodborne disease outbreaks, with *Typhimurium* as the most important serotype in outpatients with acute diarrhea [15]. Currently, SCV has been widely recognized to be part of the bacterial natural life cycle, including in *Salmonella* spp. [16]. Only a few literatures elucidated the genetic mechanisms for the development of *S. Typhimurium* SCVs by exposure to protamine or in the intracellular environment of fibroblasts. For example, mutations in the genes *lpd*, *hemL*, *aroD*, *hemA* and *ubiA* were reported to be responsible for the occurrence of *S.Typhimurium* SCVs [16, 17]. The SCVs have a slow growth rate; however, these mutants are unstable that can revert to a phenotype of rapid growth at high frequency [18]. In *S. enterica*, only one genetic basis for SCV reversion was described by Maria et al. [19]. There can be multiple pathways involved in SCV reversion, and so far for a number of *S.enterica* revertants, the mechanism of reversion is still a speculation. Accordingly, it is of importance to disclose the full lifestyles of *S. Typhimurium*, its survival strategy, and to identify the genetic changes underlying the life cycle of *S. Typhimurium* SCVs.

In addition, some bacterial SCVs, such as *Pseudomonas aeruginosa* and *S. aureus* [18, 20], showed increased biofilm formation compared to their wild-type (WT) strains. The ability to form biofilms, which contributes to antibiotic resistance and protects bacteria against suboptimal environments, makes the pathogen extremely difficult to be eradicated [21, 22]. Several studies have demonstrated biofilm formation of *S.enterica* in abiotic surfaces, plant surfaces and animal epithelial cells [23]. However, the ability of biofilm formation in the life cycle of SCVs in *S. Typhimurium* is still poorly understood.

In order to disclose the mode of *S. Typhimurium* survival after treated with STR and identify the genetic changes underlying the complete life cycle of *S. Typhimurium* SCVs, the fitness, antimicrobial susceptibility, biofilm formation, and the biofilm-related genes expression during the life cycle of SCVs were analyzed in comparison with their parent strains. Then whole genome sequencing of WT strain, SCVs, and revertants in *S. Typhimurium* was performed to analyze the genetic changes in SCVs.

## Methods

### Bacterial strains

Selection of resistant mutants was performed as described previously [24] with some modifications. Briefly, WT strain 5, a susceptible clinical isolate obtained from stool sample from an infant with acute diarrhea in Shenzhen, China, was cultured at 37 °C on Mueller–Hinton agar (MHA) added with STR (Sigma-Aldrich, St. Louis, MO) to allow cells to undergo a multistep selection process to obtain SCV strains. STR was only present in the agar during the selection, starting at 16 μg/mL and increasing twofold each step to a maximum concentration of 1024 μg/mL. A total of five *S.Typhimurium* SCVs (mutation rate: 5 × 10^−8^) were obtained in vitro. Based on the results of biofilm formation analysis, two representative SCVs (1024-4 and 1024-5) were selected for further studies. In addition, one class of fast-growing strains (approximately 10 %) was observed on antibiotic-free MHA agar after 5-day incubation at room temperature, and eight revertants reverted from strain 1024-4 or 1024-5 were collected. Two representative strains 4V1 and 5V1, which reverted respectively from 1024-4 and 1024-5, were randomly selected for further studies. All the mutants were serially passaged on *Salmonella* chromogenic agar plates (CHROMagar, France) in the absence of STR for 7 days. The stock cultures were stored at −80 °C in tryptone soy broth (TSB; Oxoid Ltd., Cambridge, UK) supplemented with 20 % glycerol until use.

### Molecular subtyping of mutants

The homology of all the mutants, including WT strain, SCVs and revertants, were identified by pulsed-field gel electrophoresis (PFGE; using XbaI) and multiple-locus variable-number tandem-repeat analysis (MLVA) following standard PulseNet protocols (http://www.cdc.gov/pulsenet/pathogens/). Dendrograms of MLVA and PFGE patterns were then analyzed using Bionumerics software (Version No. 6.0; Applied Maths, Sint-Martens-Latem, Belgium).

### Transmission electron microscopy (TEM)

Transmission electron microscopy was performed to assess the phenotypic characteristics at single cell level and the distribution of flagella, as described previously [25] with some modifications. In brief, 400-mesh carbon-coated Cu/Rh grids were incubated on 10 μL of fresh culture grown overnight for 1 min. After which, the excess media were removed by blotting off with filter paper. Each grid was dipped in 20-μL 2 % (w/v) phosphotungstic acid for 3 min. Then the grids were air-dried. To count the flagella manually, grids were examined in a TEM (Hitachi HT7700) operated at 80.0 kV and at a magnification of 10,000×.

### Fitness

In order to determine the growth of bacteria, WT strain, SCVs and revertants were incubated overnight in Luria–Bertani broth (LB) at 37 °C and adjusted to obtain the same optical density at 595 nm by fresh LB. Then, bacteria were cultured in LB medium at 37 °C for 15 h with shaking (200 r/min). The growth of bacteria in 96-well microplate was measured by Multiskan FC (Thermo Fisher Scientific Inc., USA) at 595 nm every 30 min. Each isolate was conducted with eight replicates. The broth without inoculation was used as the blank control.

### Antimicrobial susceptibility tests

Minimal inhibitory concentration (MIC) for 15 antimicrobial agents, including streptomycin (STR), imipenem (IMI), chloramphenicol (CHL), gentamicin (GEN), ciprofloxacin (CIP), trimethoprim/sulfamethoxazole (SXT), ceftriaxone (AXO), amoxicillin/clavulanic acid 2:1 ratio (AUG2), ampicillin (AMP), nalidixic acid (NAL), tetracycline (TET), cefoxitin (FOX), doxycycline (DOX), cefepime (FEP), and azithromycin (AZI), were determined by broth microdilution method using Thermo Scientific™ Sensititre™ susceptibility MIC plates (Thermo Scientific Inc., USA) following the manufacturer’s instructions. Breakpoints for sensitive, intermediate and resistant were defined by the Clinical and Laboratory Standards Institute, 2012. The *Escherichia coli* ATCC 25922 was used as quality control.

### Biofilm formation assay

Biofilm production was analyzed using crystal violet assay [26]. In passing, bacterial cultures grown at 37 °C with shaking (200 r/min) overnight were adjusted to the optical density (OD_595_) of 0.01 by diluting with TSB (1:10). Then, 100 μL of the diluted bacterial suspension was added to each well and incubated at 28 °C for 48 h without shaking. The medium was then discarded, and each well was washed with distilled water and stained with 100 μL of crystal violet for 15 min. Finally, the wells were again washed for five times with distilled water, and the biofilm formation was assayed with addition of 100 μL of 95 % ethanol to determine the OD at 570 nm by Multiskan FC (Thermo Fisher Scientific Inc., USA). The TSB (1:10) was used as the negative control. Each isolate was conducted with eight replicates.

Confocal laser scanning microscopy (CLSM) was used to study biofilm structure in mutants with the ability to form biofilm as described by Bridier et al. [27] with some modifications. Overnight bacterial cultures were adjusted to an OD_595_ of 0.01 by diluting with TSB (1:10) before being suspended in a 35-mm glass-bottom dish and incubated at 28 °C for 48 h without shaking. After the formation of biofilm, the dishes were washed five times with distilled water and refilled with diluted (1:10) TSB containing 5 μM Syto9 (1: 1000 dilution from a Syto9 stock solution at 5 mM in DMSO; Invitrogen, USA). The dishes were then incubated in the dark at 28 °C for 20 min to enable the fluorescent labeling of the bacteria. Images were acquired by CLSM (Leica microsystems Inc., Germany). Stack images were captured with a z-step of 1 μm and then biofilm images were reconstructed into three-dimensional images (3D) by IMARIS 8.0 software. The number of pixels of green fluorescent in images was determined for substratum coverage of biofilm.

### Congo red phenotype

The “rdar” (red, dry and rough) morphotype related to biofilm formation is indicative of curli fimbriae and cellulose production on Congo red plate. WT strain and all mutants were cultured overnight in TSB at 37 °C before being normalized to the same OD at 595 nm. Then, 10 μL of each culture was spotted onto an LB plate (without NaCl) containing 40 μg/mL Congo red, and further incubated for 48 h at 28 °C.

### RNA extraction and real-time PCR

RNA were extracted from WT strain, SCVs and revertants bacterial cultures grown at 28 °C for 48 h using the RNeasy Mini Kit (Qiagen; Germany), which was later used to synthesize complementary DNA (PrimeScript™ cDNA Synthesis Kit; TaKara, China). The expression levels of biofilm-related genes *csgB*, *csgD*, *adrA* and *bapA* were determined by real-time PCR following standard protocols coupled with primers described by Fa`brega et al. [24]. Relative gene expression was evaluated using the 2^−△△CT^ method, and the 16S rRNA gene was used as reference gene. Each strain was tested in triplicate.

### Whole-genome resequencing and identification of genetic variations

Genomic DNA of WT (strain 5), SCVs (strain 1024-4 and 1024-5) and revertants (4V1 and 5V1) was extracted from overnight cultures using a TIANamp bacteria DNA Kit (TIANGEN, China). Nucleic materials were sent to Beijing Genomics Institute (BGI) for whole genome sequencing using Illumina Hiseq 2000**™** platform with an average of 100× coverage per bacterial genome. Quality-filtered reads for each strain were separately aligned to the assembled contigs of WT to determine variations using SOAPaligner (v 2.21) and BWA (v 0.6.2-r126). Then the reads with indels and SNPs were mapped back to the reference genome of *S.Typhimurium* LT2 (RefSeq NC_003197.1). The indels and SNPs were further verified by PCR and Sanger sequencing method (Invitrogen, China). Also, the genetic alterations in the other revertants (strain 4V2, 4V3, 4V4, 5V2, 5V3 and 5V4) were further identified by PCR and Sanger sequencing method.

### Statistical analysis

All statistical analyses were performed using the software SPSS 13.0 for Windows as *P* value <0.05 considered to be significant.

## Results

### Phenotypic characterization of *S.Typhimurium* SCVs and revertants

**Fig. 1:**
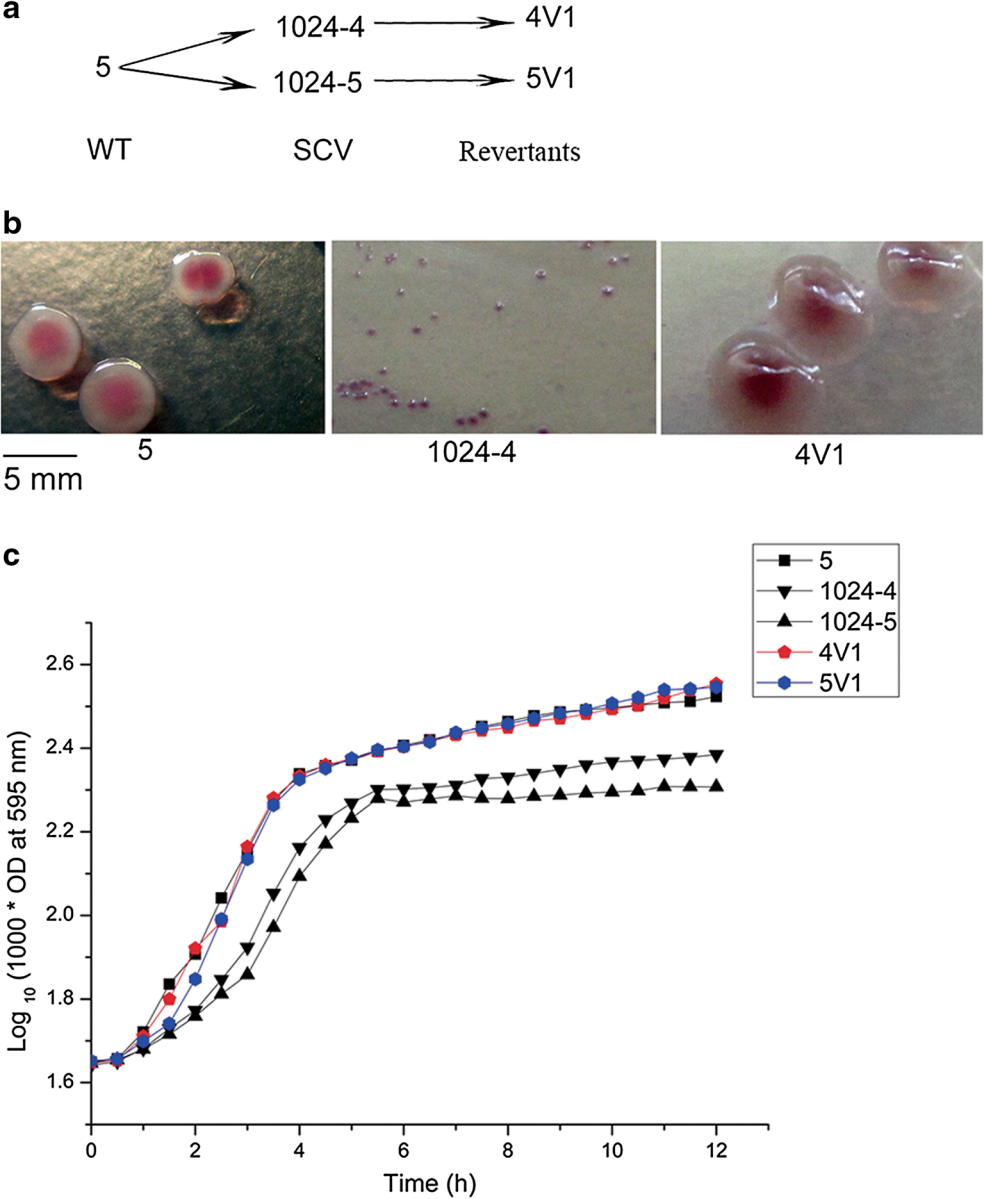
Phenotypic characterization of WT, SCVs and revertants of *S. Typhimurium*. **a** The relationship between WT (wide-type; strain 5), SCVs (strain 1024-4 and 1024-5) and revertants (strain 4V1 and 5V1). **b** Representative data on the colony morphologies of strains on chromogenic agar plate. **c** Bacterial fitness evaluated by growth curves, and strains were incubated in Luria–Bertani broth (LB) at 37 °C with shaking

One clinical isolate of *S.Typhimurium* was selected as wild-type strain, and two SCV mutants induced by STR were successfully obtained in vitro. Moreover, two fast-growing strains that reverted back from their corresponding SCVs were also obtained (Fig. 1a). WT strain and all the mutants showed identical PFGE and MLVA profiles (Additional file 1: Figure S1).

In order to study the phenotypic characteristics in the different life stages of *S. Typhimurium* SCV, the morphologies of colonies, the distribution of the number of flagella and the fitness were analyzed. The SCV colonies on *Salmonella* chromogenic agar plates appeared to be small compared with the wild-type parental strain and revertants (Representative data were shown in Fig. 1b). Based on TEM images, both strains 4V1 and 5V1 showed significantly more flagella compared to their SCV mutants (ANOVA, *P* < 0.05; Additional file 1: Figure S2). Moreover, strain 4V1 had significantly more flagella than the WT strain (*P* < 0.05). The SCVs of *S.Typhimurium* displayed slower growth rate in comparison with the WT strain, while the revertants and WT strain growing in LB obtained similar growth rate (Fig. 1c).

### Antimicrobial susceptibility test

As resistance to a variety of antibiotics is one of the typical characteristics of SCVs, we determined the MICs of several antibiotics for WT strain, SCVs and revertants (Table 1). SCVs showed two- to fourfold increments of MICs for IMI and GEN, and a drastic 64-fold increase in STR when compared with the wild-type parental strain. The revertants also showed an increase of two- to fourfold in the MICs of STR and decrease of two- to fourfold in the MICs of AMP and AZI when compared with the wild-type parental strain.

**Table 1:** MIC determination in wide-type strain (WT), small colony variants (SCV), and revertants

Strain	MIC (μg/mL)														
	STR	IMI	CHL	GEN	CIP	SXT	AXO	AUG2	AMP	NAL	TET	FOX	DOX	FEP	AZI
5 (WT)	16	<0.5	<2	<1	<0.03	<0.12/2.38	<0.25	<1/0.5	2	4	<2	4	4	<1	8
1024-4 (SCV)	>1024	1	<2	4	<0.03	0.25/4.75	<0.25	2/1	2	4	<2	4	4	<1	<2
1024-5 (SCV)	>1024	1	<2	4	<0.03	<0.12/2.38	<0.25	<1/0.5	<1	4	<2	4	4	<1	<2
4V1 (revertant)	32	<0.5	4	<1	<0.03	<0.12/2.38	<0.25	<1/0.5	<1	4	<2	4	4	<1	4
5V1 (revertant)	64	<0.5	4	2	<0.03	<0.12/2.38	<0.25	<1/0.5	<1	4	<2	4	4	<1	4

*STR* streptomycin; *IMI* imipenem; *CHL* chloramphenicol; *GEN* gentamicin; *CIP* ciprofloxacin; *SXT* trimethoprim/sulfamethoxazole; *AXO* ceftriaxone; *AUG2* amoxicillin/clavulanic acid 2:1 ratio; *AMP* ampicillin; *NAL* nalidixic acid; *TET* tetracycline; *FOX* cefoxitin; *DOX* doxycycline; *FEP* cefepime; *AZI* azithromycin

### Biofilm formation

**Fig. 2:**
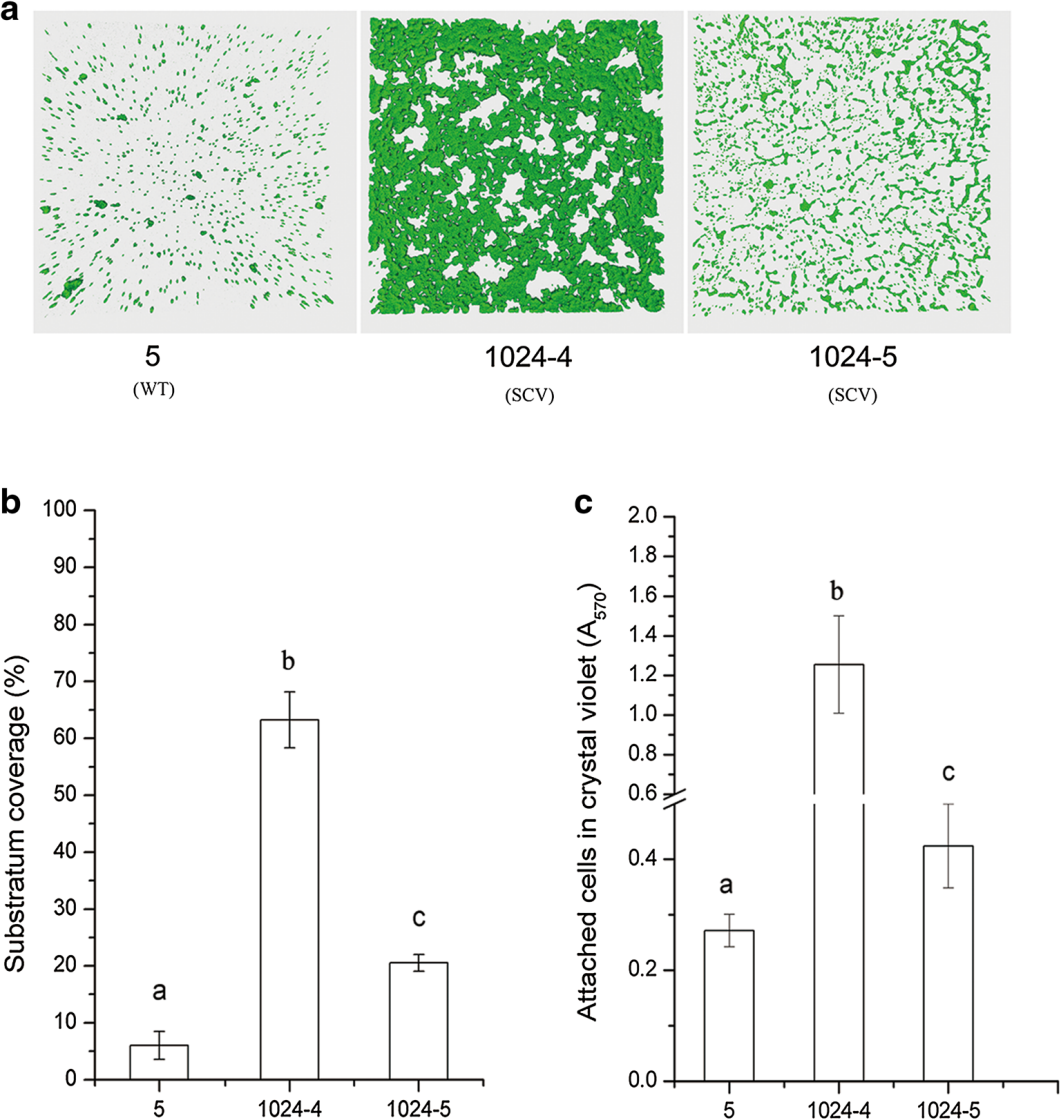
Biofilm production of WT (wide-type; strain 5) and SCVs (strain 1024-4 and 1024-5). **a** A 3D projection of a biofilm structure of SCVs obtained by CLSM. **b** Ability of biofilm formation by using substratum coverage. **c** Ability of biofilm formation based on crystal violet assay. Data are presented as mean ± SD.* Different letters* on the *error bars* indicate statistically significant differences between strains in each group based on ANOVA (*P* < 0.01)

Biofilm production was analyzed in WT strain, SCVs, and revertants (Fig. 2) and the biofilm structures of SCVs were presented in Fig. 2a. Substratum coverage of biofilm in SCVs (strain 1024-4 and 1024-5) was significantly bigger than WT strain (*P* < 0.01; Fig. 2b). Consistent with this, SCVs of strain 1024-4 and 1024-5 also exhibited significantly increased production of biofilm according to crystal violet assay (*P* < 0.01; Fig. 2c). However, this characteristic lost in all eight fast growing strains when SCVs reverted back to the normal phenotype (*P* > 0.05; Additional file 1: Figure S3).

### Expression of biofilm-related genes in SCVs

**Fig. 3:**
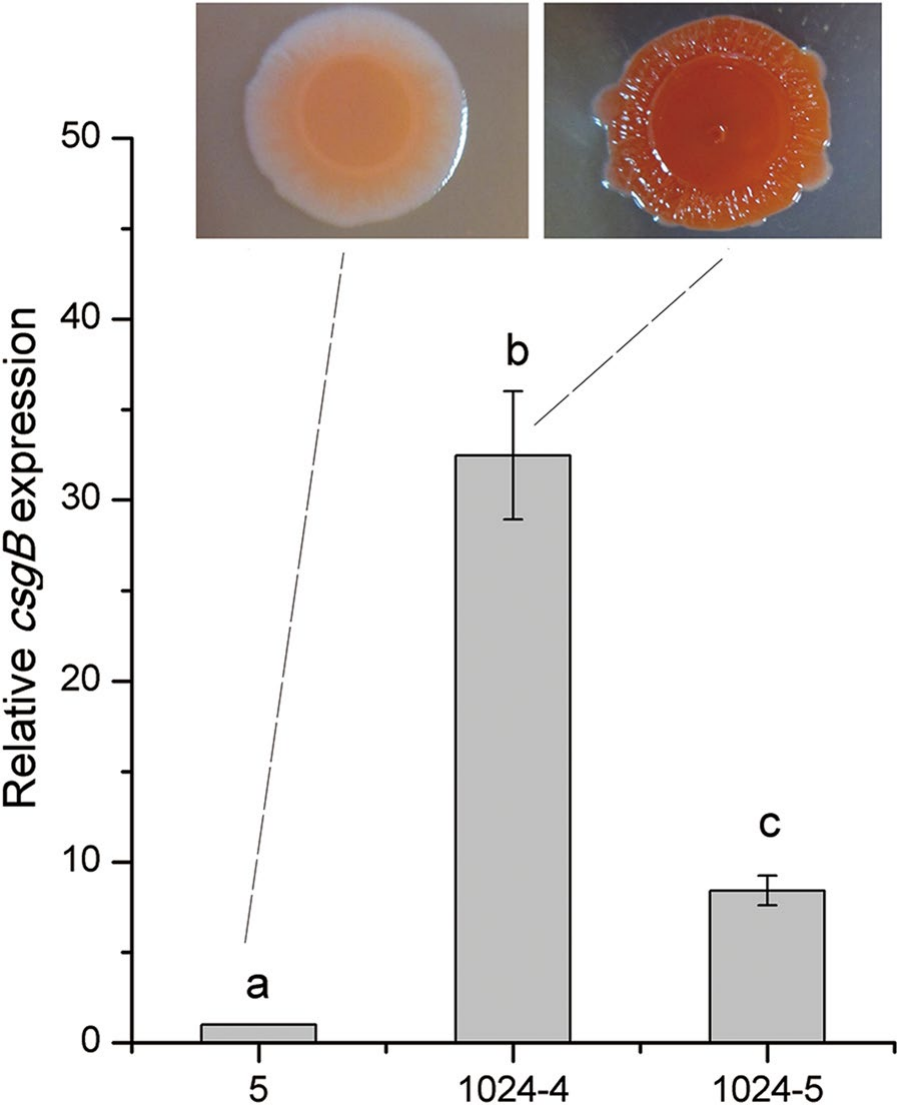
Relative expression of *csgB* gene in correlation with the rdar morphotype in SCVs (strain 1024-4 and 1024-5). Data are presented as mean ± SD. Measurement of relative expression was normalized to the WT (wide-type; strain 5).* Different letters* on the *error bars* indicate statistically significant differences between strains in each group (*P* < 0.01)

The relative expression of biofilm-related genes was analyzed in mutants with the ability to form biofilm by the RT-PCR. For strain 1024-4, expression of the gene *csgB* (*P* < 0.01) was 32 times higher (Fig. 3), while a fourfold increase was observed in both *adrA* and *bapA* genes (*P* < 0.05), and no significant change was found in *csgD* in comparison with WT strain (*P* > 0.05). For strain 1024-5, a significant increase was found in *csgB* (eightfold; *P* < 0.01), but no significant change in *adrA*, *csgD* and *bapA* (*P* > 0.05). Based on the Congo red staining, SCV strain (1024-4) showed rdar morphology which is indicative of curli and cellulose production.

### Genetic alterations

**Fig. 4:**
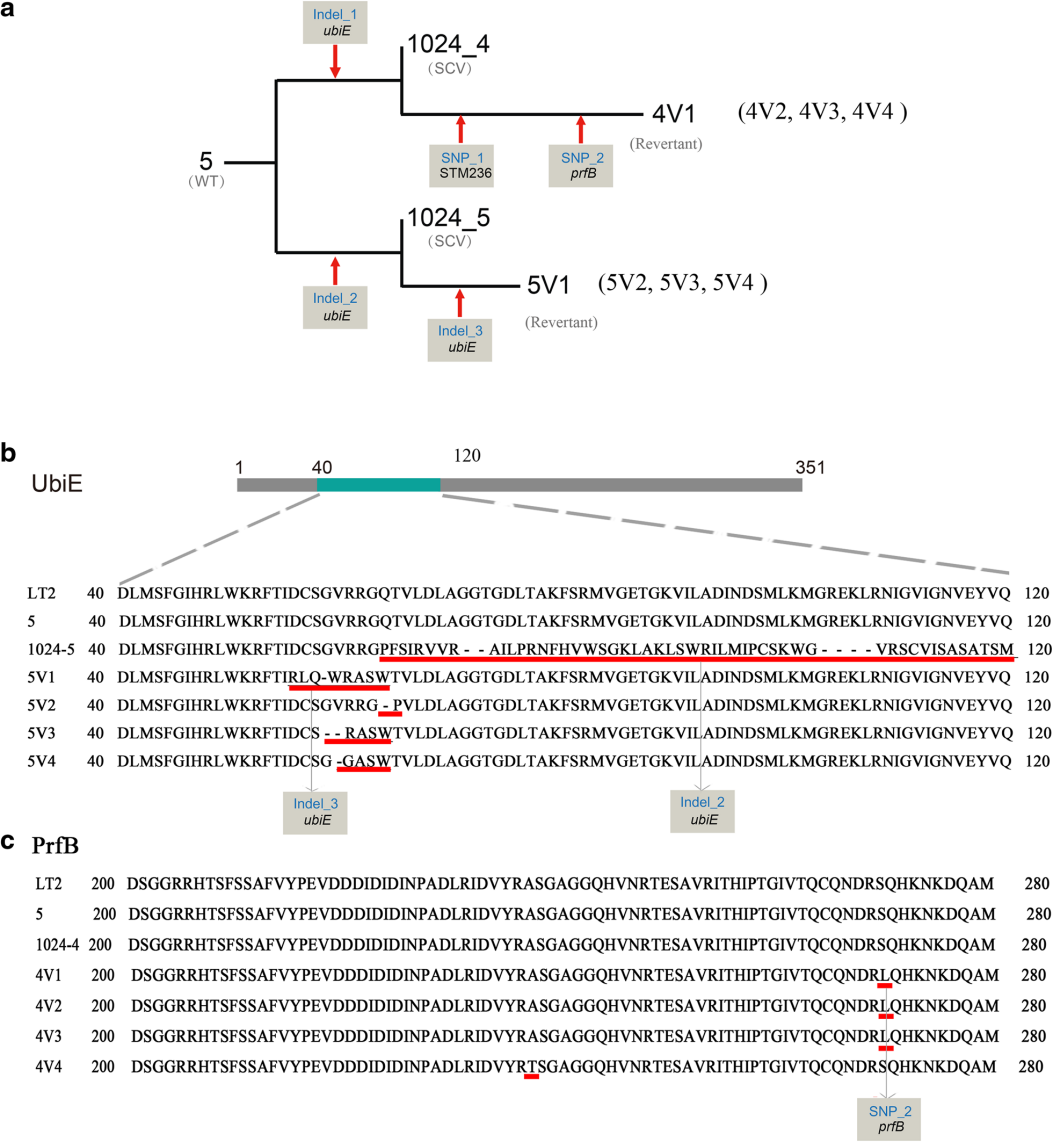
Two modes of genetic change for reversion to a rapidly growing form in SCVs. **a** Whole-genome resequencing was performed in wide-type strain (WT, strain 5), SCVs (strain 1024-4 and 1024-5) and revertants (strain 4V1 and 5V1) and the genetic alterations in the other revertants (strain 4V2, 4V3, 4V4, 5V2, 5V3 and 5V4) were further identified by PCR and Sanger sequencing method. **b** Amino acid sequence alignment of UbiE for reference strain (*S.* *Typhimurium* LT2), WT, SCVs and revertants. **c** Amino acid sequence alignment of PrfB for reference strain (*S*. *Typhimurium* LT2), WT, SCVs and revertants

To explore the genetic changes in the life cycle of SCVs, whole-genome sequences of the SCVs (strain 1024-4 and 1024-5) and revertants (strain 4V1 and 5V1) were compared to the WT strain (strain 5). Only five mutations were identified in four mutants by analyzing the data of whole genome sequencing, and notably, three of them were associated with the gene *ubiE* (Fig. 4a and Table 2). A four bp length deletion occurred in *ubiE* of SCV strain 1024-5, which might de-activate the gene through frameshift and premature termination (Fig. 4b). Interestingly, in its revertant strain, 5V1, the second mutation in *ubiE* (a one base pair insertion) recovered most of the amino acid sequence of the *ubiE* and hence might re-activate this gene (Fig. 4b). A frameshift mutation in *ubiE* was also observed in another SCV strain, 1024-4, suggesting a close association between the activity of the *ubiE* and the SCV phenotype (Table 2).

**Table 2:** List of genetic changes in *S. Typhimurium* SCVs and revertants

Mutation^a^	LT2 Position^b^	Mutant strain	Mutant type	Amino acid change	Gene name	Gene product
Indel_1	4,177,676	1024_4, 4V1	Insertion: T	Frame shift	*ubiE* (STM3970)	Ubiquinone/menaquinone Biosynthesis C-methyltransferase UbiE
Indel_2	4,177,484:4,177,487	1024_5, 5V1	Deletion: GTTT	Frame shift	*ubiE* (STM3970)	Ubiquinone/menaquinone biosynthesis C-methyltransferase UbiE
Indel_3	4,177,459	5V1	Insertion: T	Frame shift	*ubiE* (STM3970)	Ubiquinone/menaquinone biosynthesis C-methyltransferase UbiE
SNP_1	2,480,126	4V1	SNP: G to A	F125F	STM2367	Inner membrane protein
SNP_2	3,201,008	4V1	SNP: G to A	S271L	*prfB* (STM3041c)	Peptide chain release factor 2

^a^The genetic changes in SCVs and revertants were compared to the wild-type parental strain by analyzing the data of whole genome sequencing

^b^Corresponding position in the reference genome of *S.* *Typhimurium*, LT2 (Accession number: NC_003197.1)

For strain 4V1, the revertant of 1024-4, carried two SNPs beside in addition to the defective *ubiE* (Fig. 4a). One SNP occurred in a putative gene that encodes for an inner membrane protein (Table 2). However, this SNP did not change the amino acid sequence of the product, and could then just be a neutral mutation. Another SNP in the gene *prfB* led to a substitution of serine by leucine (S271L). This non-synonymous mutation could compensate the functional deficiency of mutated *ubiE*, resulting in the recovery of the phenotype (Fig. 4c).

Based on the results of PCR amplification and sequencing of mutated genes in the other revertants (5V2, 5V3, 5V4, 4V2, 4V3 and 4V4), a total of two modes of genetic change for reversion to a rapidly growing form were observed. The revertants (strain 5V2, 5V3 and 5V4) that reverted back from strain 1024-5, also harbored a secondary mutation in *ubiE* (Fig. 4b), which reinstated most of the amino acid sequence of the *ubiE*. In addition, revertants (strain 4V2, 4V3, 4V4) from 1024-4 harbored a mutation in *prfB* (Fig. 4c).

## Discussion

Slower growth, reduced metabolic activity and increased antimicrobial resistance are typical characteristics for bacterial SCVs [28], which have been most extensively studied for *S. aureus*. In this study, *S. Typhimurium* SCVs obtained by the treatment of STR also displayed slower growth rate and increased antimicrobial resistance, additionally, with partially increased production of biofilm in comparison to the WT strain.

From the perspectives of food industry, the occurrence of *S. Typhimurium* SCV may increase the potential infection risks. Since STR is still widely used in farm industry in developing countries, exposure to STR could result in the occurrence of *S. Typhimurium* SCVs. Slower growth and reduced metabolic activity are typical characteristics for SCVs of many bacteria. Conventional identification tests (i.e. biochemical tests) are hampered or modified by the decreased metabolism of these SCVs giving no reaction or false negative results [1, 29], which may complicate the routine isolation and identification of bacteria for laboratory-based surveillance of contamination in food. In this way, the risk of *Salmonella* infection might become increased.

Clinically, the phenotypes of SCVs are associated with persistent and recurrent infections in *S. aureus* and *P.aeruginosa* due to their increased ability to persist in mammalian cells and increased antimicrobial resistance [2]. In *S. aureus,* Long et al. have demonstrated that the minimum bacteriocidal concentration was two- to fourfold higher than the parental strain, which may correlate to the bacterial persister cells, however, there was very little change in MIC for SCVs [30]. Consistently, a slightly increase in MIC of IMI and GEN was observed in *S.Typhimurium* SCV mutants. In addition, these SCVs remained susceptible to critical antimicrobials, such as AUG2, AMP and CIP. These data confirm that switching to the SCVs phenotype does not bring a great change in MIC. Additionally, there was a great increase in STR resistance as these SCVs were selected by STR.

In most bacteria, the ability to form biofilm contributes to conferring tolerance and firm adhesion, which helps the bacteria to survive in suboptimal environments, making it extremely difficult to be eradicated. Therefore, the food borne pathogenic bacteria with the ability to form biofilm may threaten the safety of food industry [31]. In this study, a total of five *S.Typhimurium* SCVs were obtained, among which two mutants (strain 1024-4 and 1024-5) displayed a significantly increased biofilm formation in comparison to their wild type strain (ANOVA, *P* < 0.05; data not shown). Consistent with this, approximately 30 % of *P. aeruginosa* SCVs was observed to exhibit increased attachment to glass by Drenkard et al. [32]. Several genes, including *csgD, adrA, csgB* and *bapA*, have been identified to be associated with biofilm formation in *S. enterica* and they were quantified by RT-PCR in this study. *csgD*, a transcriptional regulator, decides whether *S.enterica* strains would form biofilms [33]. On the other hand, the gene *adrA*, which controls the levels of cyclic di-GMP, is related to cellulose production, and *csgB* is the nucleator of the curli fimbriae [34]. In addition, BapA is a large secreted protein required for biofilm formation in *S.* Enteritidis [35]. In our study, *csgB* expressed at a much higher level in SCVs, compared with the WT strain. Particularly, the relative expression of *csgB* reached a maximum of 32-fold in strain 1024-4, suggesting that “rdar” morphotype and biofilm production observed in this strain were possibly attributable to an increased expression of this gene. The associated production of cellulose and curli fimbriae have been well characterized as the “rdar” morphotype which is related to biofilm formation in *S.enterica* [36].

Coenzyme Q is a redox lipid that functions in the respiratory electron transport chain, and also plays a critical role in energy generating processes [37]. Biosynthesis of coenzyme Q in *E. coli* depends on the *ubi* genes [38]. The *ubiE* gene encodes ubiquinone/menaquinone biosynthesis methyltransferase, which is required for C-methylation reactions in both coenzyme Q_8_ and menaquinone biosynthesis [39]. In this study, a frameshift mutation in *ubiE* was observed in all SCVs, which possibly led to the deficiency of electron-transport and the formation of SCV phenotype. As previously reported, the biogenesis of flagella would be inhibited in the strains bearing deletion mutation of *ubiE* due to the lack of energy source [40]. However, complete deletion of *ubiE* is not naturally occurring in any life stages of bacteria. In this study, SCVs displayed less flagella with a mutation that naturally occurred in *ubiE*, which may be due to the reduced energy generating for flagellar biogenesis [41].

Fitness costs can be offset by compensatory mutations. In this study, two modes of the genetic changes for reversion to the rapidly growing form were observed. All the four revertants that reverted back from strain 1024-5, harbored secondary mutations in *ubiE*. It was possible that the secondary changes in the protein-coding domain led to recovery on the electron-transport compared with SCV strain (strain 1024-5). Moreover, another mode of genetic change for reversion to a rapidly growing form was observed in the revertants reverting back from 1024-4 (strain 4V1, 4V2, 4V3, 4V4). All the revertants harbored a mutation in *prfB* (encoding for peptide chain release factor 2), suggesting that the deficiency in electron-transport could be compensated by the mutation of *prfB* gene. Peptide chain release factor 2 gene, *prfB*, was catalyzes translational termination at UGA or UAA codons [42], but how the mutation in *prfB* compensated the growth cost of SCVs needs to be further investigated.

## Conclusion

To conclude, this study disclosed the specific genetic changes that involved in the life cycle of SCVs in *S. Typhimurium*, which helps to get a better understanding of the genetic basis for the ability of SCVs to revert to the phenotype of rapid growth. *S. Typhimurium* could switch to the phenotype of SCV by a mutation in *ubiE* under the treatment of STR. Moreover, the available biofilm formation of *S. Typhimurium* SCVs may be a strategy for survival. Therefore, the rational use of STR in agriculture or farm industry should be considered to reduce the potential risk of *S. Typhimurium* infection. Under a favorable environmental condition, these SCVs can escape from the growth restriction by a second-site compensatory mutation in *prfB* or a new mutation in *ubiE*. These genetic changes (SNPs and indels) that occurred in the life cycle of *S.Typhimurium* SCVs were different to those previously reported in the literature. And these findings may contribute to developing phenotype-directed treatments against *S. Typhimurium* SCVs.

## Additional file

10.1186/s12941-016-0151-3 Dendrograms of wide-type, SCVs and revertants evaluated by MLVA and PFGE. **Figure S2.** Representative data on transmission electron microscopy analysis of strain morphology and the number of flagella. **Figure S3.** Ability of biofilm formation of revertants and WT strain.

## Abbreviations

*S.**Typhimurium*: *Salmonella enterica* serotype *Typhimurium*
SCVs: small colony variants
STR: streptomycin
WT: wild-type strains
MHA: Mueller–Hinton agar
TSB: tryptone soy broth
PFGE: pulse field gel electrophoresis
MLVA: multilocus variable number tandem repeat analysis
CDC: center for disease control and prevention
TEM: transmission electron microscopy
PCR: polymerase chain reaction
SNP: single-nucleotide polymorphism
LB: Luria–Bertani broth
MIC: minimal inhibitory concentration
IMI: imipenem
CHL: chloramphenicol
GEN: gentamicin
CIP: ciprofloxacin
SXT: trimethoprim/sulfamethoxazole
AXO: ceftriaxone
AUG2: amoxicillin/clavulanic acid 2:1 ratio
AMP: ampicillin
NAL: nalidixic acid
TET: tetracycline
FOX: cefoxitin
DOX: doxycycline
FEP: cefepime
AZI: azithromycin
CLSM: confocal laser scanning microscopy
cDNA: complementary DNA
rdar: red, dry and rough
