# Supplementary material for: Phenotypic and genetic changes in the life cycle of small colony variants of Salmonellaenterica serotype Typhimurium induced by streptomycin
Source: Ann Clin Microbiol Antimicrob. 2016 May 31;15:37. doi: 10.1186/s12941-016-0151-3 (PMC4888536; doi:10.1186/s12941-016-0151-3)
Supplement: Supplementary file 1 — 10.1186/s12941-016-0151-3 Dendrograms of wide-type, SCVs and revertants evaluated by MLVA and PFGE. Figure S2. Representative data on transmission electron microscopy analysis of strain morphology and the number of flagella. Figure S3. Ability of biofilm formation of revertants and WT strain. [file 12941_2016_151_MOESM1_ESM.doc]

**Supplementary Materials for**

**Phenotypic and genetic changes in the life cycle of small colony variants of *Salmonella* *enterica* serotype Typhimurium induced by streptomycin**

Wanli Li ^1^, Yinghui Li ^2^, Yarong Wu ^3^, Yujun Cui ^3^, Yao Liu ^1^, Xiaolu Shi ^2^, Qian Zhang ^2^, Qiongcheng Chen ^2^, Qun Sun ^1^, ^#^ and Qinghua Hu ^2^, ^#^

**Affiliations:**

^1^ *Key Laboratory of Bio-resources and Eco-environment of the Ministry of Education, College of Life Sciences, Sichuan University, Chengdu, Sichuan 610064, P. R. China*

^2^ *Shenzhen Major Infectious Disease Control Key Laboratory, Shenzhen Center for Disease Control and Prevention, Shenzhen, Guangdong 518055, P. R. China*

^3^ *State Key Laboratory of Pathogen and Biosecurity, Beijing Institute of Microbiology and Epidemiology, Beijing 100071,* *P. R. China*

^#^ **Corresponding author:**

Q. Sun, College of Life Sciences, Sichuan University, 29# Wangjiang Rd., Chengdu, Sichuan 610064, P.R. China. Phone: +86-28-85418810, Fax: +86-28-85460487, E-mail: qunsun@scu.edu.cn.

Q. Hu, Shenzhen Centre for Disease Control and Prevention, Shenzhen, Guangdong 518055, China. Phone: +86-755-86580705, Fax: +86-755-25532595, E-mail: huqinghua03@163.com.

**This file includes:**

**FIG S1**

**FIG S2**

**FIG S3**

**
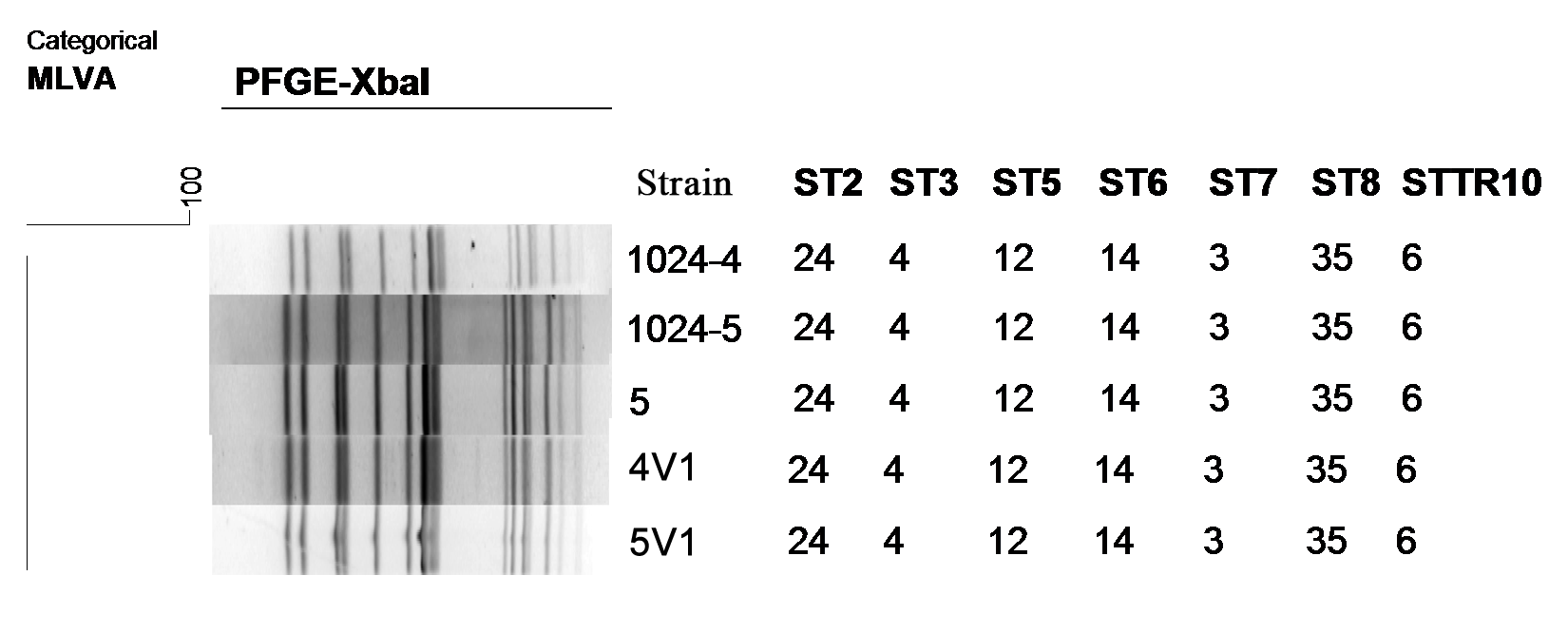
**

**FIG S1** Dendrograms of wide-type, SCVs and revertants evaluated by MLVA and PFGE. The PFGE dendrogram was generated using the Dice coefﬁcient (optimization 1.5% and position tolerance 1.5%) and unweighted-pair group method with arithmetic averages algorithm (UPGMA) algorithm. The MLVA dendrogram was generated using the categorical coefficient and UPGMA algorithm. “ST2, ST3, ST5, ST6, ST7, ST8 and STTR10” in the figure mean alleles of VNTR loci and the actual number of repeats at each locus is presented.


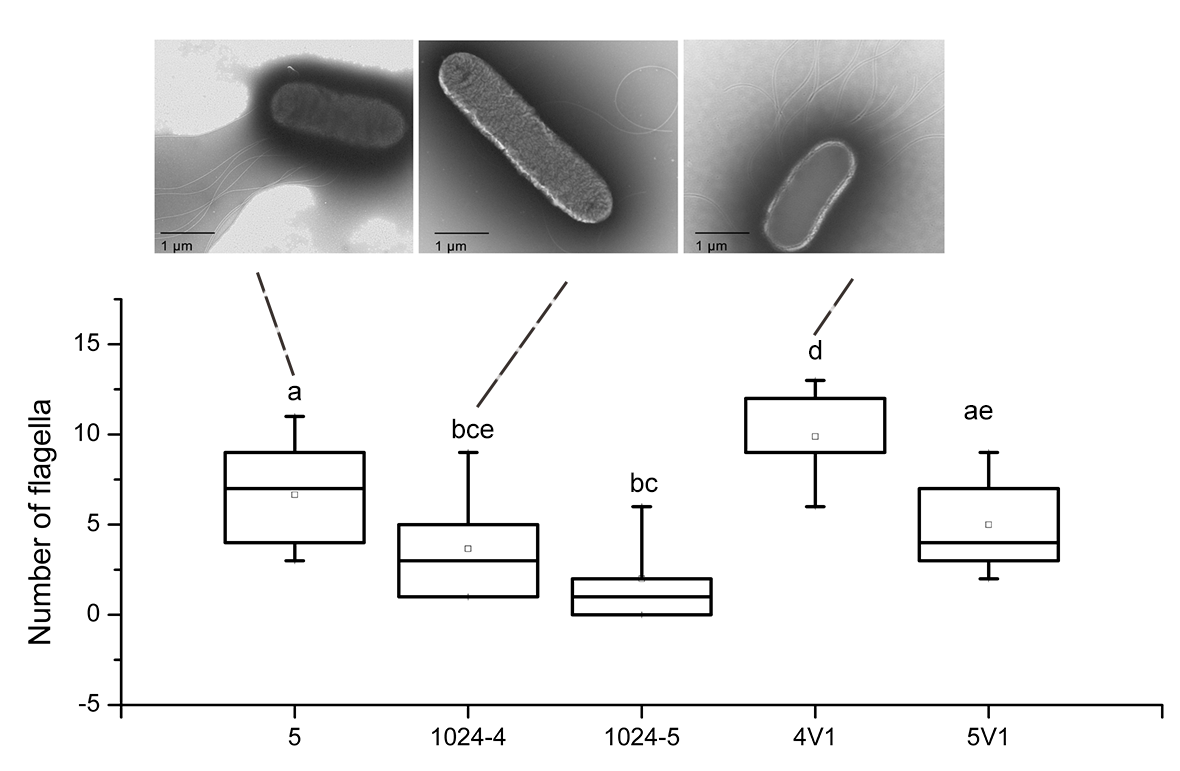


**FIG S2** Representative data on transmission electron microscopy analysis of strain morphology and the number of flagella. Different letters on the error bars are significantly different (*P* < 0.05).


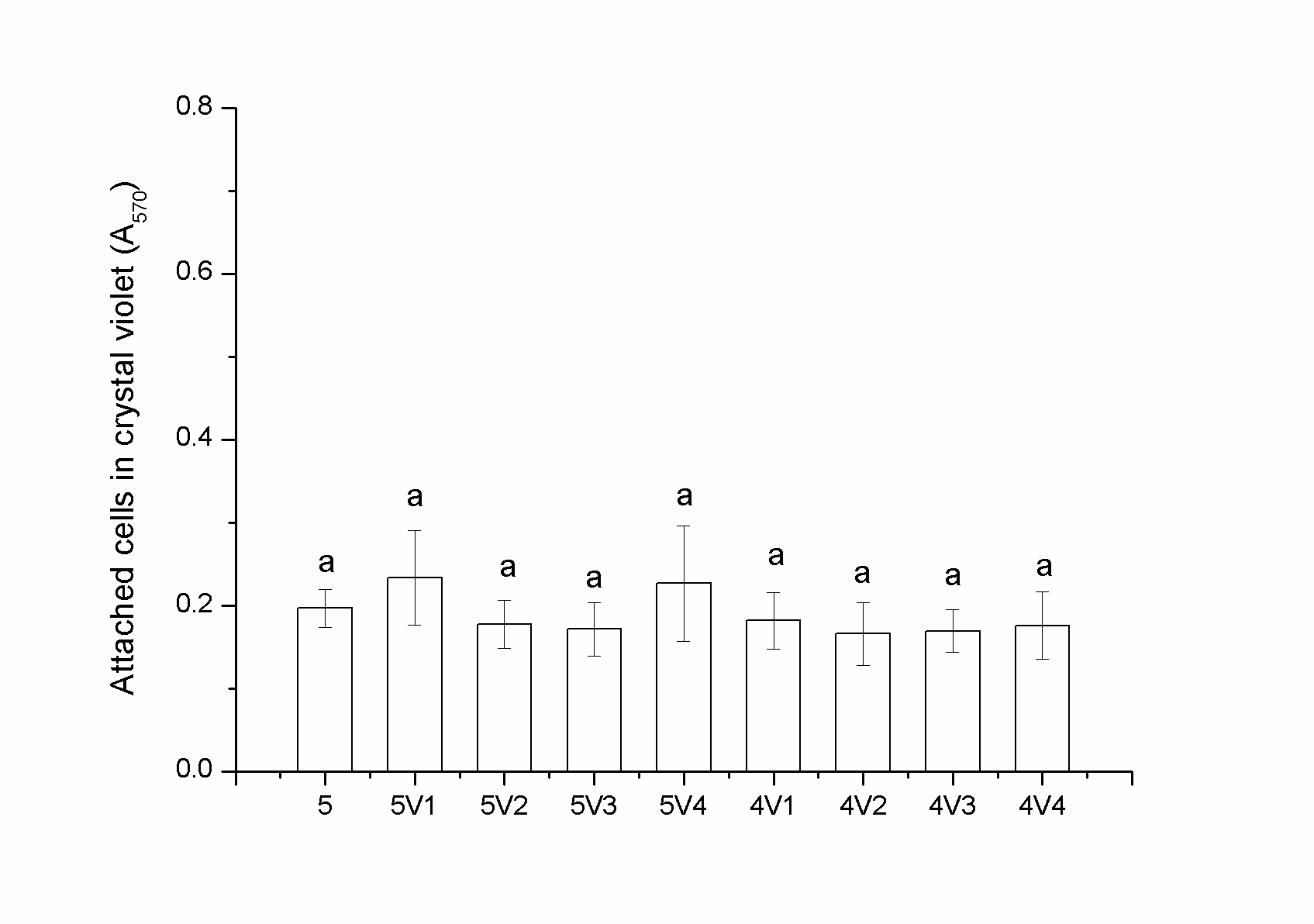


**FIG S3** Ability of biofilm formation of revertants and WT strain. “5V1, 5V2, 5V3, 5V4, 4V1, 4V2, 4V3 and 4V4” in the figure represent the strain of revertants. Data are presented as mean ± SD. Different letters on the error bars indicate statistically significant differences between strains in each group based on ANOVA (*P* < 0.05).
